# Analysis of Novel Basketball Coaches: Pedagogical Content Knowledge Using Mixed Methodology

**DOI:** 10.3389/fpsyg.2021.706276

**Published:** 2022-01-28

**Authors:** Juan Granda-Vera, Lucia Granda-Ortells, Inmaculada Alemany-Arrebola, Ángel Custodio Mingorance-Estrada

**Affiliations:** ^1^Facultad de Ciencias de la Educación y del Deporte, Universidad Granada, Melilla, Spain; ^2^Departamento de Sociologia, Universidad de Granada, Granada, Spain

**Keywords:** mixed methods research, pedagogical content knowledge, novel basketball coache, polar coordinate analysis, observational methodology

## Abstract

The aim of this article is to know the role of learning tasks within the pedagogical content knowledge (PCK) of novel sports coaches in initial stages of sports training of children/young people and their impact on their daily work. Participants are two coaches in their first or second working year. A mixed methodology was used by means of polar coordinates analysis. The results agree with previous studies that established that (1) PCK of novel coaches presents deficits in task selection and modification, (2) motivation is the key factor determining tasks selection process, and (3) they do not know how much time they should dedicate to each task. Even so, better development was found in the PCK of the coaches.

## Introduction

The concept of “practical knowledge” has been central in numerous studies. Villar (1986:25) points out that recent investigations stand up for rationalization of professional behavior in classroom, since it is the space where practicing teachers develop their theories, knowledge, and beliefs.

This research on development and construction of pedagogical content knowledge (PCK) suggests that practicing teachers have developed professional knowledge of teaching; therefore, they have learned to apply theoretical knowledge to practical situations. However, this knowledge is linked to action and personally acquired by teachers who do not only connect theory and practice, but they include beliefs, values, theories, concepts, and forms of intervention in practice. Therefore, this knowledge is not acquired in initial training, but it is related to professional practice and teaching process. Unfortunately, it is not socially acknowledged, even for practical teachers who elaborate this knowledge (Del Villar, 1993).

Teaching is a reflexive practice and, therefore, an act of knowledge, since teachers elaborate a theory about their praxis. This theory is not usually explicit, but it is guiding an action. By signifying it, practical knowledge allows understanding practice and theorizing about it. Practical knowledge is “idiosyncratic, personal, arising from one’s own experience, and delimited in its nature and extension by the characteristics of the working context; it is knowledge about practice and emerging from practice” (Zabalza, 1991, p. 123).

For its clarity and ability to synthesize other contributions, we chose the structure elaborated by Grossman (1990) who develops a scheme to explain how teachers organize their knowledge, distinguishing among (a) general pedagogical knowledge, (b) subject matter knowledge, (c) knowledge of context, and (d) pedagogical content knowledge.

Physical education content has been studied in recent years by many authors, although the term they use is “Pedagogical Knowledge of Content” (Rovegno, 1992; Granda, 1998; Castejón Oliva and Giménez Fuentes-Guerra, 2017; Cañadas et al., 2019). Some of the conclusions reached by these studies on PCK are (a) “it is very specific to the domain of the ongoing activity,” (b) “it is related to action analysis,” (c) “it undergoes a long-term evolution in relation to professional competence,” and (d) “it depends on the system limitations” (Amade-Escot, 2000).

Studying the PCK of teachers implies delving into key issues of the teaching profession, such as professional ethics, and contributing to change perception on teachers as knowledge transmitters to professionals able to apply pedagogical, disciplinary, and didactic knowledge in the classroom. This transition should be done from a reflective, critical, integrative, and complex conception of the human and educational phenomenon, favoring the discovery of new learning and teaching styles (Jimenez et al., 2017). PCK is continuously reworked during teaching action, so teachers can adapt it to context in order to create a new practical structure with important repercussions for student learning and for the teacher’s own reconfiguration of the teaching process (Vázquez-Bernal and Jiménez-Pérez, 2019).

In this sense, Cañadas et al. (2019) state that “an adequate mastery of what and how to teach the subject content, implies that teacher must design the tasks so that students learn the content, and implement activities using different techniques, styles and teaching strategies” (p. 284), so these activities are related to PCK. These decisions of teachers must emerge from the relationality of the classroom.

PCK in the field of Physical Education is controversial when determining the specific aspects related to content knowledge that a PE teacher should know. Castejón Oliva and Giménez Fuentes-Guerra (2017) state that “facing this disjunctive (knowing in depth the content and how to teach it in PE classes), it seems necessary to deepen on PE teacher’s perspective on Knowledge of Context and Pedagogical Content Knowledge at different times” (p. 147).

Among other studies on PE teachers’ PCK, Kim et al. (2018) focused on the role of learning tasks; they conducted a retrospective analysis of teachers and students’ data comparing two randomized group with one controlled quasi-experimental group, which was focused on improving PCK and student performance. Seven teachers and 32 student groups participated in this research.

PCK was measured using four variables: task selection, representation, adaptation, and an aggregate variable called total PCK. The findings of this study stated that the professional development of teachers’ content knowledge is necessary to improve the PCK of teachers and student performance (Kim et al., 2018).

Nkala and Shehu (2016) conducted a study with beginner and under-training teachers aimed at finding out how to facilitate the acquisition of general knowledge and particularly their PCK. Participants were assigned to control and experimental groups. The latter participated in a workshop aimed at fostering their PCK, while the teachers in the control group continued with their usual activity. Classroom lessons were recorded, and subsequent interviews were coded. The results showed that PCK improved significantly among the experimental groups that had attended the training workshop on their PCK.

Araújo et al. (2017) indicated that study on PCK can be useful to examine the effectiveness of Physical Education training. They conducted a study to examine the evolution of the PCK of a group of coaches under training and novice coaches over three seasons of Sport Education, and to examine the impact of protocols that had been specifically implemented to improve the PCK of coaches. Twenty-one students and one teacher from a school class in northern Portugal participated, and data were collected from multiple sources: (a) video observations of all lessons, (b) field notes, and (c) pre-lesson interviews with coaches.

Among the most relevant conclusions after the first season, error diagnosis, feedback, task modification, and deficit in task presentation were remarkable. After the end of the third season, better development of the PCK of the coaches was found, revealing the importance of coach education protocols when designing Sports Education.

### Toward the Use of Mixed Methods Research

In Behavior and Social Sciences, and particularly in Education Sciences, there are intensive and frequent debates posed in dichotomous terms about the most appropriate methods for the development of knowledge, finding contrary positions between qualitative and quantitative advocates (Jacobs, 1989). Although this dichotomy is central in the discussion among researchers in the field of Education, the need to overcome it is becoming increasingly evident (Medina, 1992, p. 10).

According to Alvarez (1986, p. 13) “there is not an exclusive method for scientific research or science” and “both perspectives are necessary and can work together by complementing each other.” Therefore, combining methods comes up as a solution to overcome this antagonism, and it has already been suggested by many researchers in the field of Behavioral and Social Sciences (Granda, 1998; Cameron, 2009; Cabrera and Rosales, 2017). Mixed methods offer different possibilities in studies that focus on daily or spontaneous behaviors in natural contexts (Portell et al., 2015; Vázquez-Diaz et al., 2019), such as teaching behavior during a Physical Education class.

The mixed methods approach involves the collection, analysis, and interpretation of qualitative and quantitative data for the same purpose and within the framework of the same study.

Qualitative and quantitative data can be combined in three different ways, as Creswell and Plano Clark (2007, p. 7) summarized: “There are three ways in which we can mix data: merging two data sets by bringing them together, connecting two data sets using one of them as a reference, or incorporating one data set within the other so that one type of data provides a supporting structure for the other data set.” For our proposal, we chose the second option. According to Sandelowski et al. (2009), this connection can be achieved through transformation, i.e., quantifying qualitative data or qualifying quantitative data.

Collecting information about human behavior by analyzing texts and transforming it into properly systematized and organized categorical data without missing key information is a great challenge in Behavioral and Educational Sciences. There are multiple situations where the information coming from systematic observation is irreplaceable, such as information that arises from observation of the interaction between teachers and students during Physical Education classes (García-Fariña et al., 2018).

Indirect observation is an appropriate method for studying both verbal behavior and textual material by analyzing transcripts and original material produced by participants. Verbal behavior conveys a wide range of messages that can be transmitted using different channels. Thus, messages are analyzed differently depending on whether they are spoken or written.

Indirect observation is considered a valid scientific method using similar techniques to systematic observation and presenting an identical structure, although there are important differences related to the nature of the source data (verbal behavior and text). Indirect observation involves the analysis of a textual material generated indirectly from transcriptions of audio recordings of verbal behavior in natural settings (e.g., conversation, group discussions) (Anguera, 2017).

Data collected during indirect observation invariably emerge as qualitative data, and the source material varies according to the level of involvement of the observed person and the source nature (textual or non-textual).

Based on these data, the objective of this study is to explore how learning tasks influence PCK and their impact on the daily teaching of novice basketball coaches.

## Materials and Methods

### Design

This research applies a mixed methodology using data collected through direct and indirect observations (Anguera et al., 2018).

It was a nomothetic investigation, as there were more than one participant, point because a follow-up record was applied over eight sessions (one per week to each participant for 2 months), and multidimensional because there were several levels of response (five dimensions with different categories collected through an *ad hoc* instrument developed for this study).

### Participants

The coaches who participated in this study were María and Joseph, who are novice coaches specializing in basketball (first or second training year). Both were committed to the subject and showed interest throughout their initial training period, so they met the ideal characteristics for selection.

Likewise, their great capacity for analysis and clarification in group discussions and for debating in working groups with other professionals of the field was also valued, since these abilities were considered essential for reporting on issues relevant to the study. Finally, we would like to highlight their willingness and interest in participating in the research.

### Instruments

According to Sánchez-Algarra and Anguera (2013), in systematic observation, a distinction is made between recording instruments (i.e., those used to record or code data) and observational instruments (instruments specifically designed to analyze a certain topic).

### Recording Instruments

To ensure the validity of the data, we carried out triangulation procedures in data collection using the following instruments:

(1). Participant observation: the performance of the coaches was observed, and field notes were taken throughout practice sessions. Moreover, audiovisual recordings of these sessions in the research setting provide the researcher with the opportunity to re-witness the content. Erickson (1990) calls this recording process “microethnography.” It should be noted that technological records are not sufficient and they cannot replace field notes, but rather they are an additional source of data apart from direct observation.
  These recordings are also used to make the participants relive the significant events related to the object of study, so they can remember and explain the thoughts that guided their actions, as well as the opinions, judgments, beliefs, and knowledge that influenced or justified such actions.
(2). Interviews to stimulate recall: a total of eight semi-structured interviews were conducted over the research period with every participant. The video recordings of each of the participants during their performance in class were used as a reference for the interviews. The interviews were conducted on the same day of the recording using as a semi-structured script of the field notes taken during participant observation. The interview was selected as an instrument because “the interview is based on the idea that teachers/coaches are able to offer an explanation of their behavior, their practices and their actions to those who ask them about them” (Walker, 1989, p. 125).
(3). Diaries of participants: The contributions of the coaches were collected in personal diaries during the research.

### Observation Instruments

The *ad hoc* observation instrument used in the study was transformed into a category system instrument in which the different dimensions were divided into different categories according to theoretical framework and experience (see Annex 1).

All the texts were transcribed and coded using the category system shown in Annex 1. The AQUAD 8.0 software package was used for the whole process. Finally, polar coordinate analysis was performed to analyze the data extracted from the reduction of the texts, a process named “liquefaction” by Anguera (2020).

### Procedure

The teachers participating in the study were informed about the objective of the study, and both accepted to collaborate in the research. They were contacted based on the data obtained through observation in the training course and the practice session carried out afterward.

All practice sessions were recorded in video, and the interviews were conducted immediately after each practice session was totally transcribed. There were 8 1-h sessions over a 2-month period, and one session was conducted per week. Along with these sessions and subsequent interviews, the coaches kept a diary throughout these 2 months in which they wrote down issues related to their teaching that they found significant. In addition to these 8 sessions, a final interview was conducted and included in the final analysis. Each participant performed 8 interviews in addition to the final interview.

All the interviews and diaries were categorized by assigning each piece of text a code according to a previously designed category system, allowing for the qualitative data to be transformed into quantitative data.

Quantification is essential in the mixed methods framework to move on to the second phase in the QUAL-QUAN-QUAL cycle (Anguera, 2020). Afterward, code matrices are quantitatively analyzed, which enables the connection of functions and integration of qualitative and quantitative values, so qualitative data can be analyzed quantitatively after the transformation process (Anguera et al., 2018).

### Data Quality Control: Interobserver Agreement

For data quality control analysis, four of the interviews were analyzed and coded by two observers who had been previously trained using the approach described by Anguera (2003). Before recording the data, the observers agreed on the category or code to which each of the observed actions was assigned (consensual agreement).

Agreement was quantitatively assessed using Cohen’s kappa statistic (Cohen, 1968). The level of agreement was “almost perfect,” with kappa values ranging from 0.85 to 0.9 for all the sessions.

### Data Analysis

Data were constructed by assigning paragraphs to codes specified in the category system. A polar coordinate analysis was conducted, which is considered a categorical data reduction technique that would allow us to construct a vector image of the complex network of interrelationships between codes of the observation instrument. In this research, we used the categories of the subdimensions as codes for this procedure. The HOISAN program (Hernández-Mendo et al., 2012) was used for the polar coordinate analysis.

The structure of the polar coordinate analysis, proposed by Sackett (1980), complements the prospective (forward) (Bakeman and Quera, 2011) and retrospective (backward) (Anguera, 1997) perspectives of the lag sequential analysis carried out from a series of sequentially recorded codes. In this study, the codes correspond to the textual units of each of the participants throughout the interviews and diaries. Anguera and Losada (1999) suggest analyzing at least five prospective and five retrospective lags (−5 to + 5).

This technique provides interpretable data through the application of an extremely powerful data reduction algorithm based on the Zsum statistic, introduced by Cochran (1954). Sackett (1980) applied the Zsum statistic to perform both prospective and retrospective calculations. Precisely, he applied it to obtain adjusted residual values considering the criterion behavior of the sequential analysis as the focal behavior and conditional behaviors in positive lags to obtain prospective Zsum values. Later, he did the same with negative lags to obtain retrospective Zsum values. Sackett (1980) recommended using the same number of prospective and retrospective lags.

Associative relationships are sought between one code (known as focal behavior) and the rest of the codes (conditional behaviors) to perform this analysis. In this study, the codes were categories of the dimensions described in our category system. The focal behavior selected was TAR (classroom activities) following the studies conducted by Kim et al. (2018) and Cañadas et al. (2019).

**FIGURE 1:**
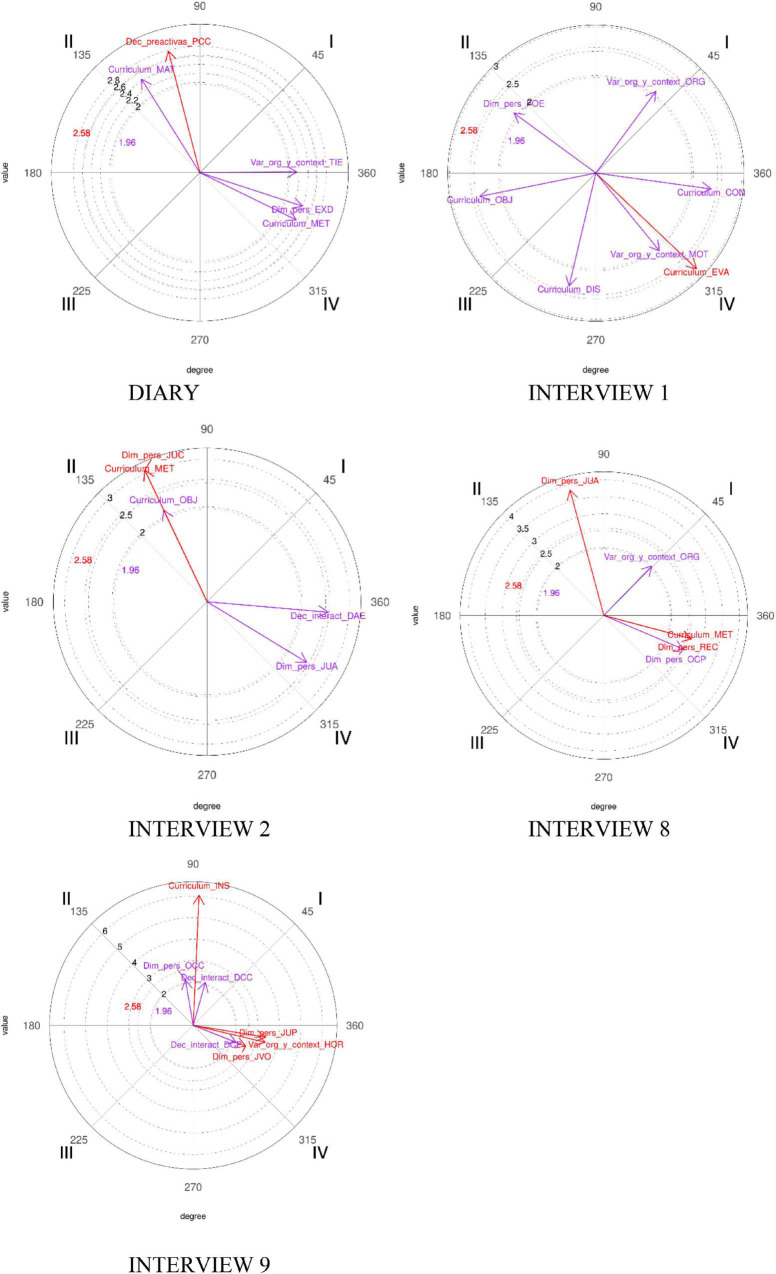
Graphics showing the relationships between Joseph’s focal behavior (TAR) and conditional behaviors.

The relationship between the focal behavior and each of the conditional behaviors is depicted by the length and angle of the corresponding vectors shown in a graphical polar coordinate map whose interpretation is shown in Figure 1 (quadrant I (0 < φ < 90) = φ; quadrant II (90 < φ < 180) = 180 − φ; quadrant III (180 < φ < 270) = 180 + φ; quadrant IV (270° < φ < 360°) = 360° − φ) and whose interpretation is presented in Table 1 (Arias-Pujol and Anguera, 2020).

**TABLE 1:** Interpretation of polar coordinate maps.

Quadrant	Sign of Zsum prospective	Sign of Zsum retrospective	Interpretive meaning
I	+	+	Focal and conditional behavior activate each other
II	−	+	The focal behavior inhibits the conditional behavior, which activates the focal one
III	−	−	Focal and conditional behavior mutually inhibit each other
IV	+	−	The focal behavior activates the conditional behavior, which inhibits the focal behavior.

These vectors depict the complex network of interactive associations among behaviors, both quantitatively (length of the vectors) and qualitatively (angle of the vectors).

Of all the interviews, only those that showed significant relationships between the task category and the rest of the categories were considered for inclusion in the subsequent analysis.

Learning tasks (LTs) were considered as focal behavior, and the rest of the tasks were defined as conditional behaviors. This choice is justified by the fact that in PCK studies, tasks are considered the key element of curriculum by teachers and coaches (Zabalza, 1991). We present in the next sections the results of the polar coordinate analysis (see Tables 2, 3).

**TABLE 2:** Polar coordinate analysis results for the relationship between the focal category TAR and Joseph’s diary and interviews.

Category	Quadrant	ProspectiveP.	RetrospectiveP.	Ratio	Radius	Angle
**DIARY**						
Curriculum_MET	IV	2.14	–1.05	–0.44	2.38 (*)	333.88
Curriculum_MAT	II	–1.29	2.07	0.85	2.44 (*)	121.95
Dec_preactive_PCC	II	–0.7	2.69	0.97	2.78 (**)	104.51
Var_org_and_context_TIE	I	2.15	0.01	0.01	2.15 (*)	0.38
**INTERVIEW 1**						
Curriculum_DIS	III	–0.55	–2.31	–0.97	2.38 (*)	256.66
Curriculum_OBJ	III	–2.39	–0.48	–0.2	2.43 (*)	191.39
Curriculum_CON	IV	2.37	–0.33	–0.14	2.39 (*)	352.11
Curriculum_EVA	IV	2.06	–1.96	–0.69	2.85 (**)	316.44
Var_org_and_context_ORG	I	1.23	1.67	0.81	2.08 (*)	53.71
Var_org_and_context_MOT	IV	1.31	–1.6	–0.77	2.06 (*)	309.28
Dec_preactive_PCC	III	–0.15	–2.46	–1	2.46 (*)	266.54
**INTERVIEW 2**						
Curriculum_OBJ	II	–0.92	1.93	0.9	2.14 (*)	115.42
Curriculum_MET	II	–1.31	2.77	0.9	3.07 (**)	115.33
Dim_pers_JUC	II	–1.31	2.77	0.9	3.07 (**)	115.33
**INTERVIEW 8**						
Curriculum_MET	IV	2.59	–0.69	–0.26	2.68 (**)	345.06
**INTERVIEW 9**						
Curriculum_INS	I	0.27	6.04	1	6.05 (**)	87.43
Dec_interact_DCP	IV	1.99	–0.78	–0.37	2.14 (*)	338.49
Dec_interact_DCC	I	0.56	2	0.96	2.08 (*)	74.25

*Focal behavior: TAR.*

*Conditional behavior: The rest of the categories.*

*p < 0.05, **p < 0.01.

**TABLE 3:** Relationships between the TAR category and the rest of the categories in the diary and interviews of Maria.

Categories	Quadrant	ProspectiveP.	RetrospectiveP.	Ratio	Radius	Angle
**DIARY**						
Curriculum_CON	II	–1.44	1.35	0.68	1.97 (*)	136.98
Var_org_and_context_ESP	I	0.34	2.37	0.99	2.4 (*)	81.84
Dim_pers_COD	IV	2.69	–1.28	–0.43	2.98 (**)	334.48
Dec_preactive_PCC	II	–1.73	1.8	0.72	2.49 (*)	133.83
**INTERVIEW 1**						
Curriculum_MET	III	–1.91	–1.95	–0.71	2.73 (**)	225.6
Curriculum_TAR	I	2.72	2.72	0.71	3.84 (**)	45
Curriculum_EVA	III	–0.96	–1.72	–0.87	1.97 (*)	240.8
Dim_pers_COD	IV	1.59	–1.44	–0.67	2.15 (*)	317.79
Dec_interact_DCC	IV	3.04	–0.94	–0.29	3.18 (**)	342.85
**INTERVIEW 4**						
Curriculum_CON	IV	3.38	–0.55	–0.16	3.42 (**)	350.79
Var_org_and_context_TIE	II	0.78	2.05	0.93	2.2 (*)	110.84
Dec_interact_DCC	I	2.52	0.21	0.08	2.53 (*)	4.8
**INTERVIEW 6**						
Curriculum_TAR	I	1.87	1.87	0.71	2.65 (**)	45
Curriculum_EVA	IV	1.88	–1.37	–0.59	2.33 (*)	323.89
Var_org_and_context_MOT	III	–2.16	–1.92	–0.67	2.89 (**)	221.74
Dec_preactive_PCC	I	2.51	0.41	0.16	2.54 (*)	9.29
**INTERVIEW 7**						
Var_org_and_context_ESP	II	–0.47	2.14	0.98	2.19 (*)	102.31
**INTERVIEW 8**						
Curriculum_OBJ	I	3.14	1.56	0.45	3.51 (**)	26.49
Curriculum_MAT	I	3.83	1.19	0.3	4.01 (**)	17.3
Dec_preactive_PCC	I	4.35	2.23	0.46	4.89 (**)	27.11
**INTERVIEW 9**						
Curriculum_CON	III	–2.67	–0.46	–0.17	2.71 (**)	189.83
Dim_pers_COD	I	2.18	1.96	0.67	2.93 (**)	41.95

*Focal behavior: TAR.*

*Conditional behavior: The rest of the categories.*

*p < 0.05, **p < 0.01.

## Results

**FIGURE 2:**
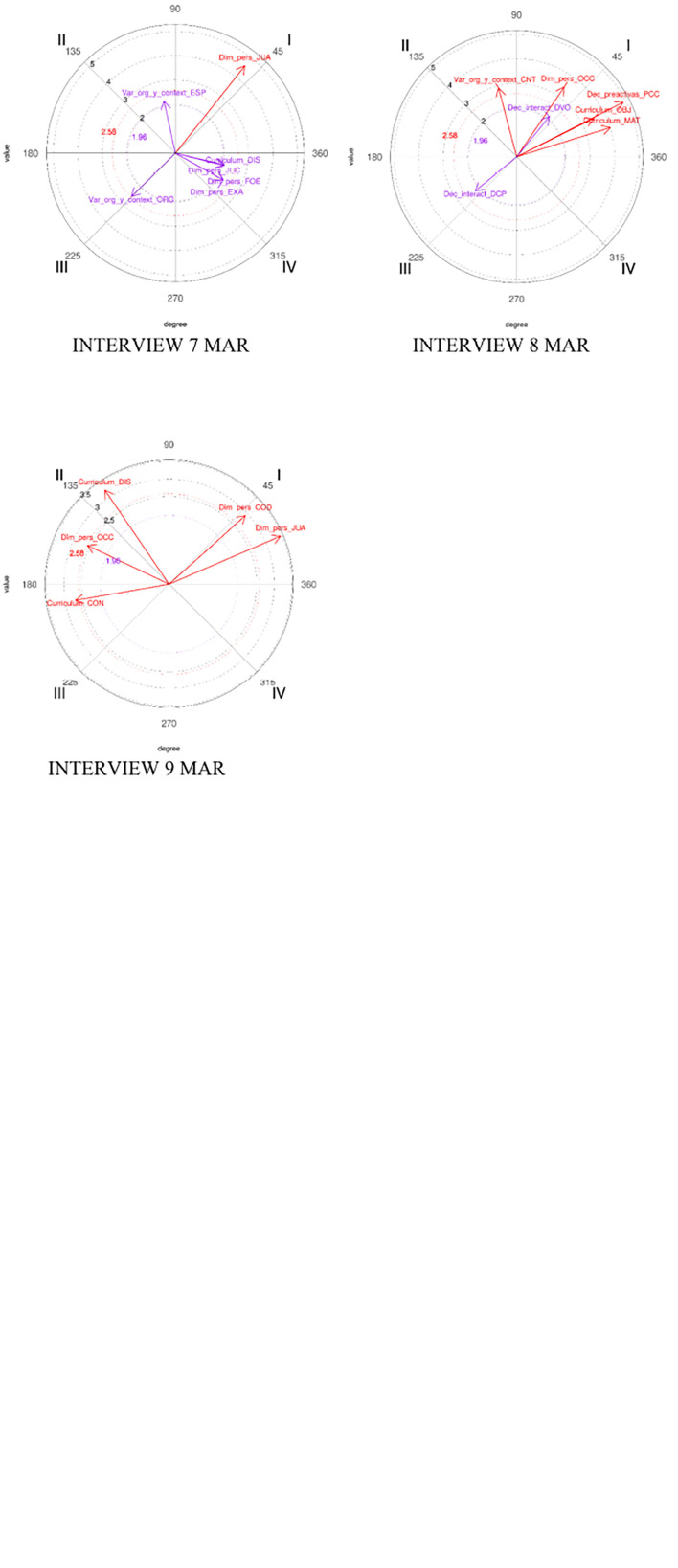
Graphics showing the María’s relationships between focal behavior (TAR) and conditional behaviors.

In the following sections, we describe the existing relationships among the categories that were directly related to PCK in the analysis, although more relationships have emerged from the polar coordinate analysis (see Figures 1, 2). A relationship is considered statistically significant when the value of the vector is equal to or greater than 1.96.

### Joseph’s Results

Table 2 shows the significant relationships between learning tasks (TAR), which was considered the focal behavior, and Joseph’s diary and interviews, which were taken as conditional behaviors.

The results reveal significant relationships between the TAR category and methodology (MET), and preactive decisions (PCC) and objectives of the curriculum (OBJ), on more than one occasion over time, while the rest of the behaviors do not present any relationship throughout the research.

Regarding methodology, Figure 1 shows that relationships evolve from a position where the tasks are activated (dependence) by the methodology and the tasks do not show any influence on the methodology (quadrant II), to a position in quadrant IV, where the learning tasks activate the use of a certain methodology without conditioning task structure.

As for preactive decisions (PCC), in early phases of the learning process, the relationships between TAR and PCC categories show that the tasks inhibit curriculum decisions in the preactive phase of the teaching/learning process (quadrant II) and activate the learning tasks. Subsequently, these relationships evolve to mutual inhibition (no active relationship) in quadrant III.

In the relationship between TAR and OBJ, initially, these two categories inhibit each other (quadrant III), evolving toward a relationship in which tasks are not related to learning objectives but to other learning variables; objectives were selected according to the learning tasks implemented by Joseph in the classroom.

The relationships between TAR and O do show total interdependence between them (quadrant I), which reveals Joseph’s interest in task organization and selection of tasks according to their organization.

Initially, the duration time of the tasks shows a high relationship with the tasks, and they influence each other; it reveals a high concern between learning time needed to acquire the content presented in the task and interest shown by athletes/students when approaching it. Finally, the mutual interest between tasks and task duration disappears.

The relationship between the level of athletes/Students’ motivation (MOT) when approaching a task is also a relationship of great concern in the early stages of the training process, since the degree of motivation has a considerable impact on the tasks (quadrant IV).

Figure 1 shows the graphs with vectors representing the relationships between TAR and the other behaviors of Joseph, and how they evolve over time (change among the different quadrants). The figures show all the significant relationships found, although they have not been incorporated into the final analysis, because they are not related to the objective of this article.

### Maria’s Results

Table 3 shows the significant relationships between learning tasks (TAR) and the diary and interviews of Maria.

Maria shows a greater number of relationships between the focal behavior (TAR) and the rest of the behaviors than Joseph. From all the relationships, this research focuses only on those more relevant to PCK and learning tasks.

The results show that TAR and teaching content (CON) appear in quadrants II, III, and IV, which means that tasks inhibit the content, and that the content influences the design and selection of the tasks. It evolves toward a position where tasks activate (influence) content (she selects the tasks; according to these tasks, she selects the content they address). Finally, no mutual influence is observed in the relationship, since they mutually inhibit each other.

As for the connection between TAR and her behavior as trainer (COD), the results reveal that tasks have a great influence on it, evolving as the learning process goes on toward a relationship of mutual influence, which indicates the importance that the tasks have for Maria as a coach (mutual activation between both categories).

The relationships between ART and preactive decisions (PCC) show that in the early stages of the training process, tasks inhibit curriculum decisions in the preactive learning phase (quadrant II), activating the learning tasks. These relationships evolve to mutual activation (quadrant I), being taken into consideration when planning the session and influencing the planning of the tasks.

Regarding evaluation (EVA) (assessment of their behavior of their athletes), the relationship between TAR and EVA evolves as time goes by; the tasks are not considered initially in the evaluation, but finally the tasks influence (activate) the evaluation although the latter does not influence the type of tasks selected for the session.

The relationships between the tasks and the decisions made during the practice session to modify some curricular element (DCC) reflect a clear evolution. It starts from a position in which the tasks activate the decisions made in the session, although these will not affect the tasks (inhibition), to a relationship in which both categories activate each other, with the tasks being also a curricular element that can be modified throughout the practice session.

The relationships between TAR and the action space where the task is performed (ESP) also change from a position where task and space are readjusted (mutual activation), to a position where only the space activates the task and modifies the structure of the tasks.

Regarding the results of Maria, the relationships between TAR as focal behaviors and TIE and MOT as conditional behavior do not present the same directionality; duration time influences the task (quadrant II), and TAR and MOT are located in quadrant III (mutual inhibition quadrant).

Figure 2 shows the graphs with vectors representing the María’s relationships between ART as the focal behavior and the conditional behaviors, and how they evolve over time (change among the different quadrants). The figures show all the significant relationships found, although they have not been included in the final analysis, because they are not related to the objective of this article.

## Discussion

The aim of this study was to find out the role of learning tasks in relation to PCK and what influence they have on their daily practice in novice basketball coaches.

The results present data reduced through an analysis of polar coordinates without missing relevant information about the behaviors of the coaches related to learning tasks and the rest of the behaviors, or the impact of these factors on the PCK of the novice coaches.

The results reveal that the novice coaches consider students, tasks, and instructional environment or context as key elements in their teaching process.

The results indicate that PCK development led to discernment of content knowledge in relation to the interactions among new tasks, new environments, and new capabilities and intentions of athletes/students (Rovegno, 1992).

The dynamics among athletes/students, learning tasks, and learning environment is, thus, the fundamental unit of how PCK is developed.

Moreover, evidence supports that tasks are the basic unit of planning (Tillema, 1984), and it is confirmed that learning tasks, both in the preactive phase (task design) and during the practice session (interactive phase), are central in the learning process of athletes/students.

This selection of learning tasks is not based on the analysis systems leaned during their previous training, but they used as criteria the educational potential of the tasks (the possibility for athletes/students to acquire the teaching contents), their own convictions, and level of the athletes/students and their motivation.

These criteria are presented without clearly stating which elements and references are used to establish the adequacy or inadequacy of the tasks with respect to these elements of analysis, assuming an initial lack of analysis and reflection that would have avoided this problem.

I also realize during the realization of the activity that the partners organization had not been adequate because of the difference in height between the partners, so they could not balance each other because of the difference in weight, and when I realized that and I have modified the partners (JOSEPH, DIARY).

The structural potential of the tasks and what they can teach at the time of selection are factors considered in their selection.

Q: Why did you select this game?

A: It is a game where you work on displacement

I like this game because it highlights very much how they have to solve the fact of being chasers and not being able to catch. (JOSEPH, INTERVIEW 2).

The factor “level of motivation” is crucial, since the participants in this study pointed out that involving athletes/students in the learning tasks is extremely difficult if they do not have fun during the practice, giving importance to the factor “motivation” over any other element both in the task selection and design process and in the performance of these tasks. Moreover, María noted that, although motivation was the main criterion, the age of the students and available material and space were also considered.

The changes that I introduced were related to organization (individual, pairs and trios). The last two options were more motivating for the students (MARY, DIARY).

This approach reinforces the motivation of and interest of the students/athletes during their activity in the training session.

I look for other strategies but as I see that they are working well and they like it, which I think is important, that they like what they do, so I am working it this way (JOSEPH, INTERVIEW 8)

This suggests that this decision may be influenced by his interest in ensuring that the proposed tasks motivate their students.

As for the TAR-TIE relationship, this is conditioned by the degree of interest and participation of the students. The duration of the classroom tasks is not predicted in the preactive phase, and with this behavior being usual among beginner Physical Education teachers; they struggle to calculate the necessary time for each task, and on many occasions, students do not have time enough to learn and adequately retain the main points of new skills (Smytj, 1992).

Regarding the duration of each task, practice time, and learning time, they point out that they do not plan them, emphasizing that it is a decision they make according to their previous experience as players. They use as criteria to establish the duration of each one, level of activity or involvement of the athletes/students in the tasks, and their motivation.

on top of the mats and jump into free spaces. Throughout the activity the placement of the mats was varying. This activity turned out to be quite motivating, more than I expected, so I let them doing the activity longer (JOSEPH, INTERVIEW 2).

Besides, they also struggle to understand task progression and identify the tasks that would be adequate and motivating for their students. Thus, they progressively learn in every session, and they adapt content and instruction for the next practice according to the experience learned in the previous one (Graham et al., 1993).

A: Sincerely, you know many of these things as you advance in experience, as you set up activities, what problems they may involve, how you solve them. At the beginning you should make a contingency plan about what things can happen to you, what things can’t, but a lot of times there are so many unexpected events that you can’t control them, that they come up depending on the group of athletes, depending on the situation, and that is like a knowledge that you accumulate and when you have to do any plan, you think about what happened to you and you know how to control those variables and you have don’t have so many surprises over time (MARY, INTERVIEW 7).

He incorporates modifications depending on how the sessions evolve and would improve the initial proposal.

Q: It is noted in your diary that you normally carry out few tasks per session, and you usually leave a few undone, what do you think is the reason?

A: I always organize a wide range of activities and especially this usually happens to me more with games because of the variants I find, that the children have come up with or that the children or the ones that I see that can improve the game (JOSEPH, INTERVIEW 9).

Granda and Canto (1997) found that beginner coaches and teachers assume some ideas on type of athletes/students and training context that initially led to frustration and discouragement. These feelings are manifested both at the level of the relationships with their athletes/students and training environment, as well as in relation to the improvement of the teaching-learning process during the performance of tasks and in the teaching methodology (assumption of methods that are not adequate for them).

The latter is determined by teaching contents, learning tasks, and possibilities of action presented by athletes/students when they respond to proposals. In most of the didactic situations proposed, he has been able to work with the methodology with which he is most identified as a trainer.

Q: How do you select or determine the methodology to use in the session?

A: The methodology varies a lot depending on the type of content and the possibilities of the athletes, although I haven’t had any problem and I have been able to work most of the time with the methodology with which I feel more comfortable, problem solving, perhaps because it is the one I have gone in depth in my initial training, so perhaps I feel I am inclined toward this type of methodology because, as I have already said, I feel identified with it. (JOSEPH, INTERVIEW 8).

Regarding the evaluation of the learning process and athletes/students (understanding as evaluation the degree of effectiveness and efficiency they show during task performance), they decided to carry it out from the very beginning using diverse and varied situations, which allowed them access to data they consider relevant for every evaluation task.

quite specific I did not want to push them in the level of demand but rather that each one set it for themselves, providing a range of opportunities. I also did this to explore how they worked in this type of situation. I already have commented that in these first weeks I am going to have a lot of variety and to know the group (MARY, INTERVIEW 6).

This process helps him to know the level of student and identify the most appropriate tasks to propose in the sessions depending on their characteristics.

Also the initial evaluation that I have been doing during the first few months has helped me to determine what kind of activities I can and can’t do, and e.g. I’ve seen that the games that require the children to throw and catch while they are moving are very difficult for the children (MARY, INTERVIEW 9).

The results of data analysis showed that coaches’ PCK variables, including task selection, task adequacy, and task adaptations, can be turned from immature into mature depending on the CK of coaches (Kim, 2011; You, 2011).

Besides, the results confirmed that the CK of beginner coaches is still not well established, pointing out that proposing tasks and processes focused on individualized learning during the session is closely related to the structure and components of the teaching contents proposed in the previous planning.

This relationship between learning tasks and teaching contents favors or complicates these processes, missing the structural components of different teaching contents in their selection and design at different levels of curricular planning.

On the other hand, this selection is characterized for being global, open, and sometimes ambiguous, which makes two processes difficult: selection of tasks, which does not aim at teaching and learning specific components but at global improvement of content, and learning evaluation, which implies high complexity when establishing criteria to guide teachers on the performance levels achieved by their students.

## Conclusion

The main conclusions obtained from this study point out, on the one hand, and in accordance with Cañadas et al. (2019), that coaches should design tasks to facilitate Students’ acquirement of content and implement activities according to different techniques, styles, and teaching strategies. In the case of Joséph and María, they select the teaching methodology guided by the teaching content and tasks. This methodology selection is influenced by knowledge gaps of the content, not adequately associating a certain type of teaching content with the appropriate methodological approach.

In the case of Joseph and Maria, task selection is revealed as an evidence-based practice to improve the PCK of coaches/teachers, as indicated by Kim et al. (2018).

Finally, the most relevant conclusions were the deficit in task presentation and task modification, although better development of the PCK Of the coaches was found, as Araújo et al. (2017) asserted.

Practical implications derived from the findings of the present study would be aimed at encouraging reflection by coaches during their training period. This reflection can be developed through discussion in small groups (using a seminar model) or protocols that motivate them to consider the difficulties encountered to facilitate their access to PCK and to become more involved in their professional development as coaches.

As for limitations of this study, the small number of participants limits the possibility of generalizing these results to all basketball coaches during their training and first years of experience, although this circumstance does not undervalue the importance and acquisition of knowledge of the findings.

## Data Availability Statement

The original contributions presented in the study are included in the article/supplementary material, further inquiries can be directed to the corresponding author/s.

## Ethics Statement

The studies involving human participants were reviewed and approved by the Ethics committee of Facultad de Ciencias de la Educación y del Deporte (Universidad de Granada). The patients/participants provided their written informed consent to participate in this study.

## Author Contributions

JG-V and LG-O conceived of the presented idea. LG-O developed the theory and performed the computations. IA-A and ÁM-E verified the analytical methods. JG-V investigated (a specific aspect) and supervised the findings of this work. All authors discussed the results and contributed to the final manuscript.

## Conflict of Interest

The authors declare that the research was conducted in the absence of any commercial or financial relationships that could be construed as a potential conflict of interest.

## Publisher’s Note

All claims expressed in this article are solely those of the authors and do not necessarily represent those of their affiliated organizations, or those of the publisher, the editors and the reviewers. Any product that may be evaluated in this article, or claim that may be made by its manufacturer, is not guaranteed or endorsed by the publisher.

## Annex 1

### System of Categories Used in the Research

#### Dimensions, Categories, and Codes

##### Curriculum Components

DIS: Planning for teaching-learning process
OBJ: Objectives
CON: Contents
MET: Methodology
INS: Information for students
MAT: Materials
TAR: Classroom activities
EVA: Evaluation
NEE: Curricular adaptations for students with special educational needs

##### Organizational and Contextual Variables

ORG: Group organization
CNT: Classroom control
ESP: Spaces where class takes place
HOR: Class schedules
TIE: Time dedicated to each learning task
EPA: Previous PE experiences of students
RAT: Relationship of the Students’ group with the classroom tutors
MOT: Students’ motivation

##### Personal Dimension

REL: Relationships with students
EXD: Teaching experience
FOE: Initial training
EXA: Experiences as student
OCV: Opinions on beliefs and values
OCC: Opinions on curriculum
OVO: Opinions on organizational and contextual variables
OCN: Opinions on non-curricular issues
OCP: Opinions on problematic issues
JUC: Judgment on a curricular component
JVO: Judgment on organizational and/or contextual variables
JUP: Judgment on a problematic situation
JUA: Judgment on students
DIL: Dilemmas
REF: Reflection on their teaching performance
REC: Reflection on curricular components
REV: Reflection on organizational and/or contextual variables
COD: Teaching behavior and attitudes toward teaching

##### Preactive Decisions

PCC: Decisions related to a curricular component
PVO: Decisions related to organizational or/and contextual variables

##### Interactive Decisions

DCP: Decisions to face conflict in the classroom
DCC: Decision related to a curricular component
DVO: Decision related to organizational or/and contextual variables
DAA: Decision based on Students correct performance on tasks
DAE: Decision based on Students’ erroneous performance on tasks
